# Supplementary material for: The effect of the dispersal kernel on isolation-by-distance in a continuous population
Source: PeerJ. 2016 Mar 29;4:e1848. doi: 10.7717/peerj.1848 (PMC4824897; doi:10.7717/peerj.1848)
Supplement: Figure S1 — The dispersal function, range, and probability density function for σ = 1. [file peerj-04-1848-s001.pdf]

	Probability Density Function (PDF)	PDF when $\sigma = 1$
Rayleigh	$f(r,\theta;\sigma) = \frac{1}{2\pi}\frac{r}{\sigma^2}e^{\frac{-r^2}{2\sigma^2}}$ $r \geq 0$	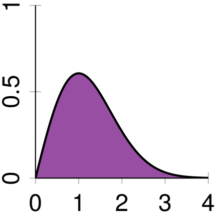
Exponential	$f(r,\theta;\sigma) = \frac{1}{2\pi}\frac{1}{\sigma}e^{\frac{-r}{\sigma}}$ $r \geq 0$	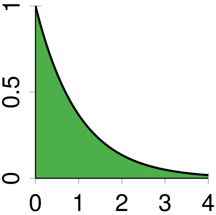
Half-Normal	$f(r,\theta;\sigma) = \frac{1}{2\pi}\frac{1}{\sigma\sqrt{\pi}}e^{\frac{-r^2}{4\sigma^2}}$ $r \geq 0$	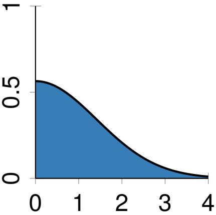
Triangular	$f(r,\theta;\sigma) = \frac{1}{2\pi}\frac{r}{2\sigma^2}$ $0 \leq r \leq 2\sigma$	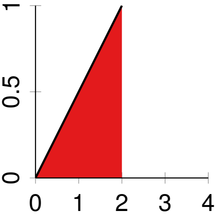
Gamma	$f(r,\theta;\sigma,\alpha) = \frac{1}{2\pi}\frac{\left(\frac{\sqrt{a(a+1)}}{\sqrt{2}\sigma}\right)^{\alpha}}{\Gamma(a)}r^{\alpha-1}e^{-r\frac{\sqrt{a(a+1)}}{\sqrt{2}\sigma}}$ $\alpha = 1, 2, 4, 8; \quad r \geq 0$	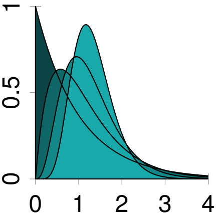
Lomax	$f(r,\theta;\sigma,\alpha) = \frac{1}{2\pi}\frac{\alpha\left(1+\frac{r}{\sqrt{(\sigma^2(\alpha-2)(\alpha-1)}}\right)^{-(\alpha+1)}}{\sqrt{\sigma^2(\alpha-2)(\alpha-1)}}$ $\alpha = 2.4, 2.6, 2.8, 3.0; \quad r \geq 0$	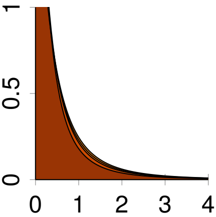
Pareto	$f(r,\theta;\sigma,\alpha) = \frac{1}{2\pi}\frac{\alpha\sqrt{\frac{2\sigma^2(\alpha-2)}{\alpha}}}{r^{a+1}}$ $\alpha = 2.4, 2.6, 2.8, 3.0; \quad r \geq \sqrt{2\sigma^2(\alpha-2)/\alpha}$	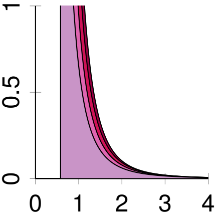
