# Supplementary figures and images for: The effect of the dispersal kernel on isolation-by-distance in a continuous population
Source: PeerJ. 2016 Mar 29;4:e1848. doi: 10.7717/peerj.1848 (PMC4824897; doi:10.7717/peerj.1848)

Empirical Cumulative Distribution Function

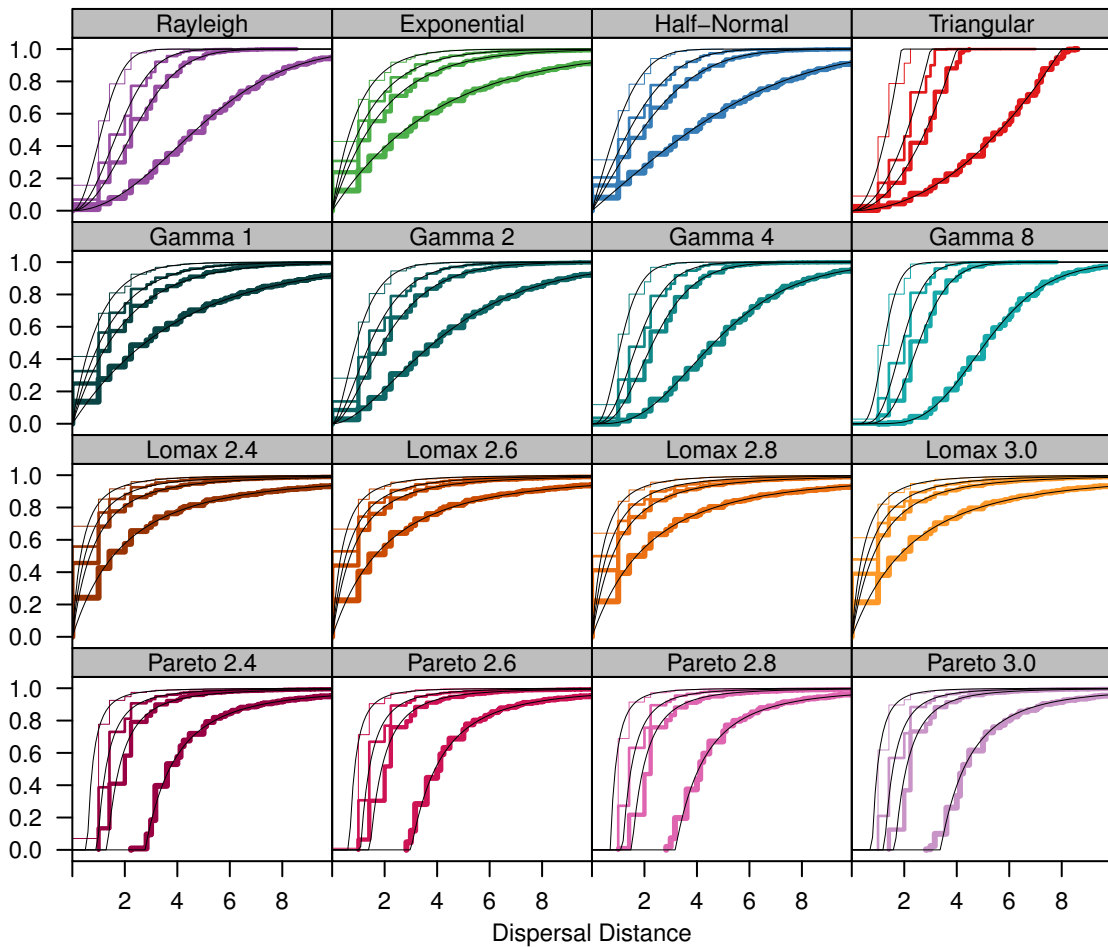

Supplement: Figure S2 — The empirical cumulative distribution function for each dispersal distribution on a discrete lattice compared to the CDF of its continuous counterpart (black line). The different plots in each panel represent simulations run using different σ parameters: 1, 1.5, 2, 4. An increase in the thickness of the line corresponds to increasing σ parameter. [file peerj-04-1848-s002.pdf]

A

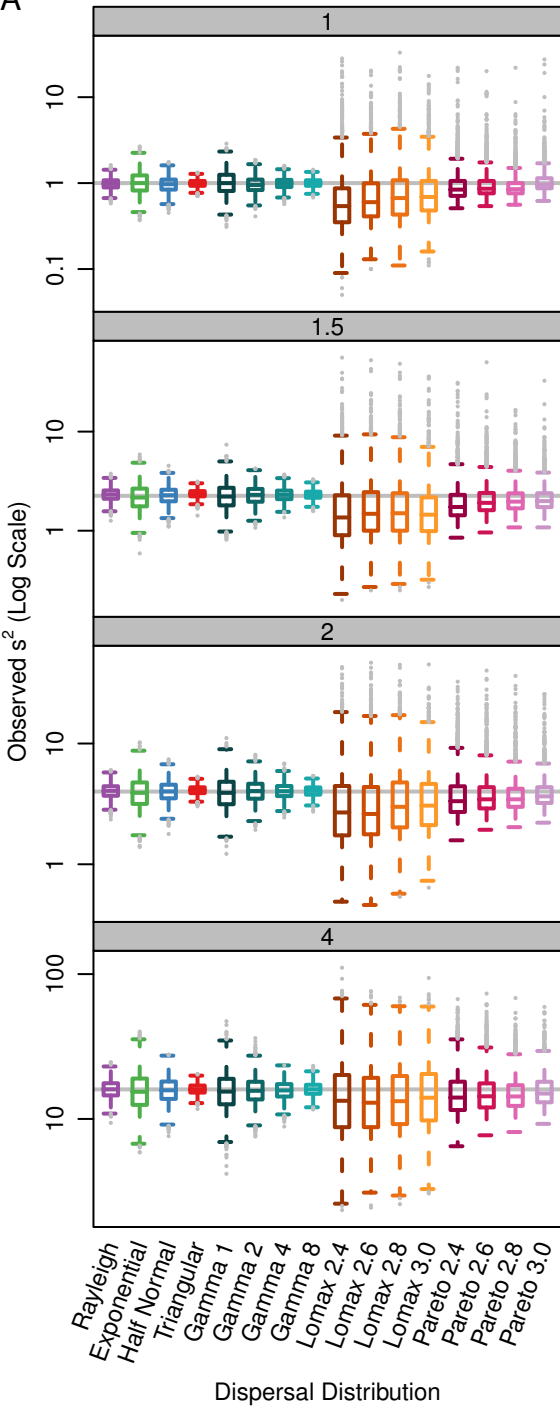

B

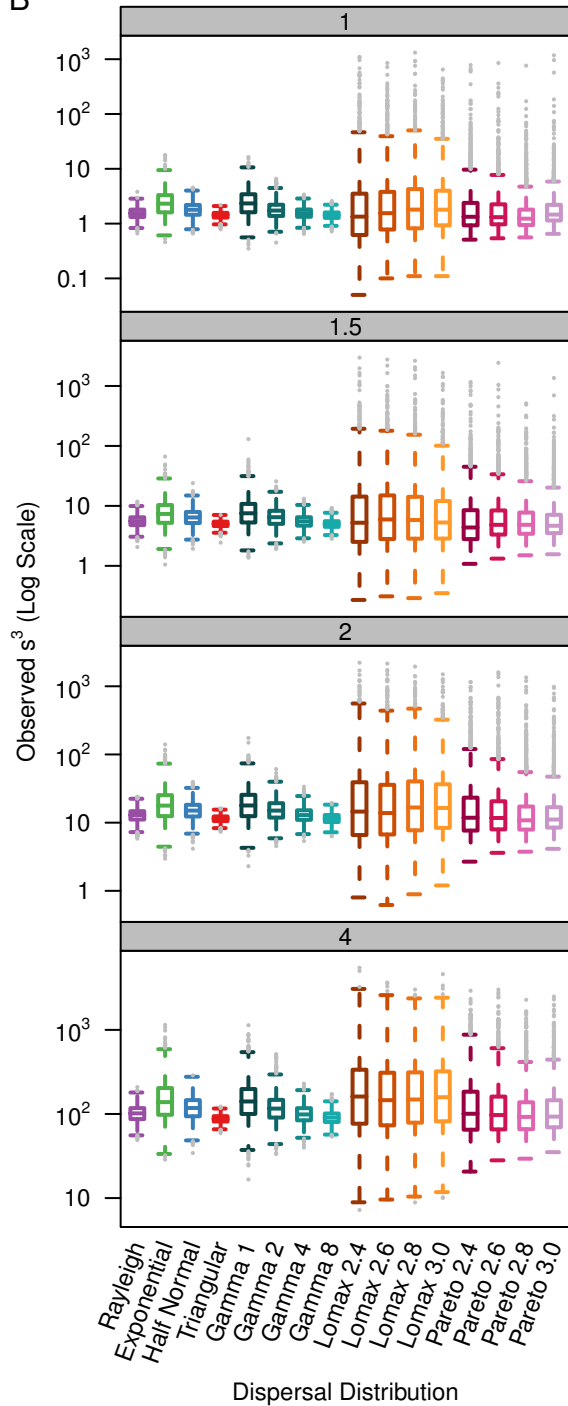

Supplement: Figure S3 — Each panel represents groups of simulations run with different σ parameters and contains box-whisker plots summarizing the distribution of the average (A) squared or (B) cubed parent-offspring distance of 2,000 sampled transects. The top and bottom of the boxes represent the 75% and 25% quartiles, while the central bar represents the median. The gray dots outside the whiskers represent outliers. The gray horizontal line in A represents the expected σ2 value. The observed values are shown on a log scale which is different in some panels. [file peerj-04-1848-s003.pdf]

Number of Alleles

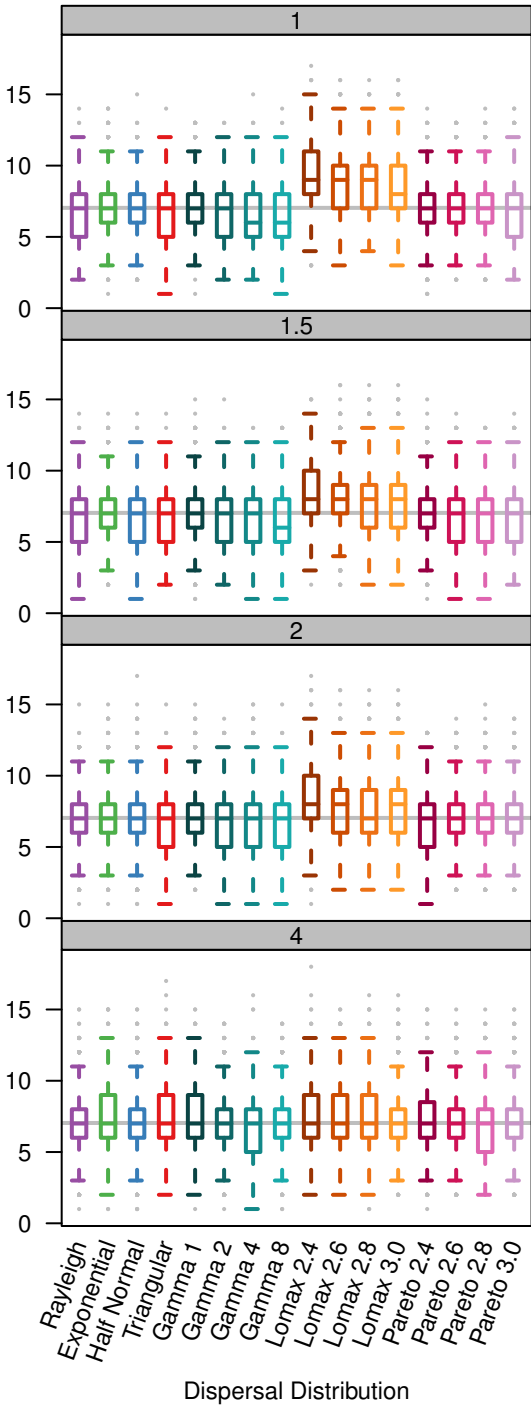

Supplement: Figure S4 — Each panel represents simulations run with a the σ parameter provided in the gray box. For each dispersal distribution, the box-whisker plot summarizes the number of unique alleles (k) found in 2,000 50-individual transects. The gray horizontal line represents the expectation under the infinite alleles model. The features of the box-whisker summary are the same as Fig. S3. [file peerj-04-1848-s004.pdf]
